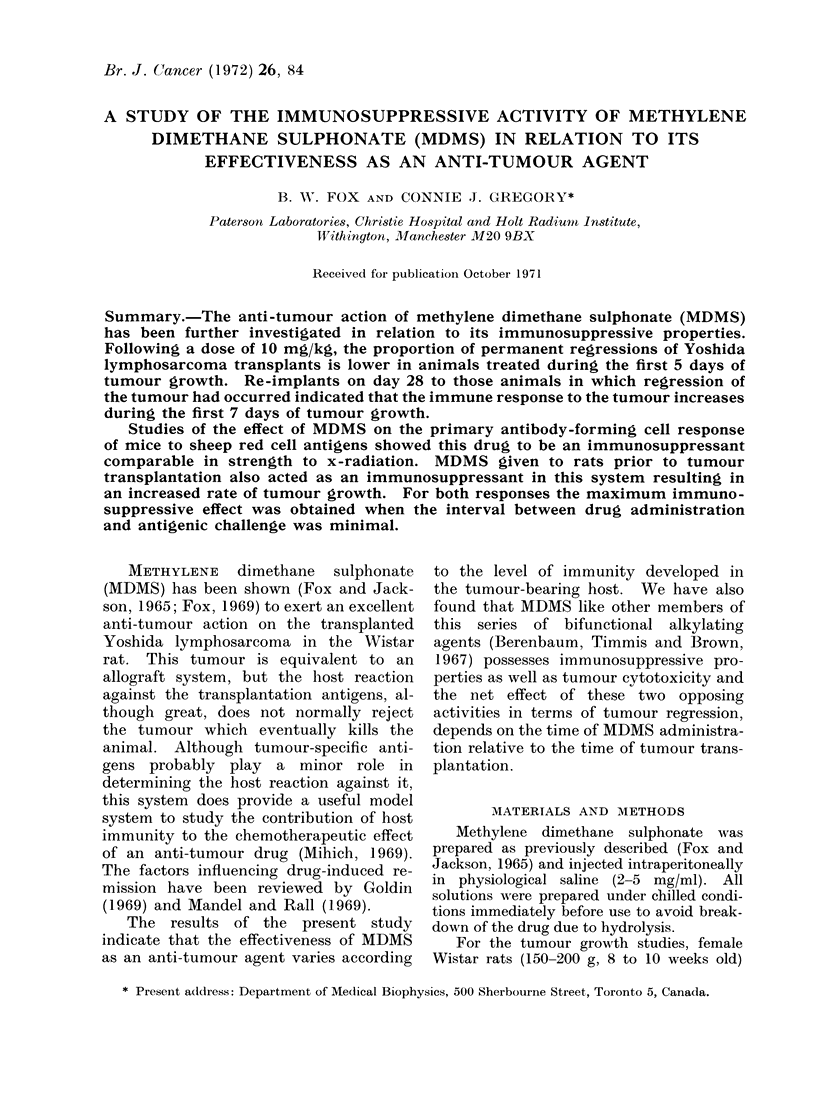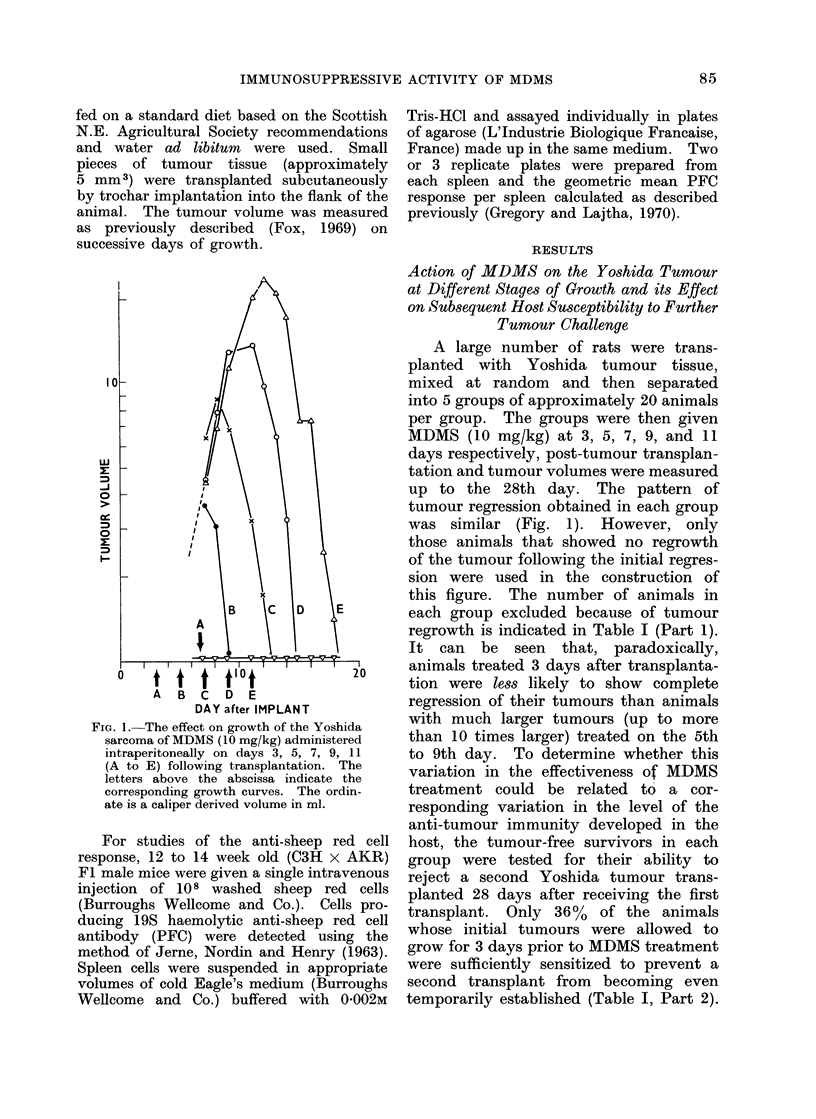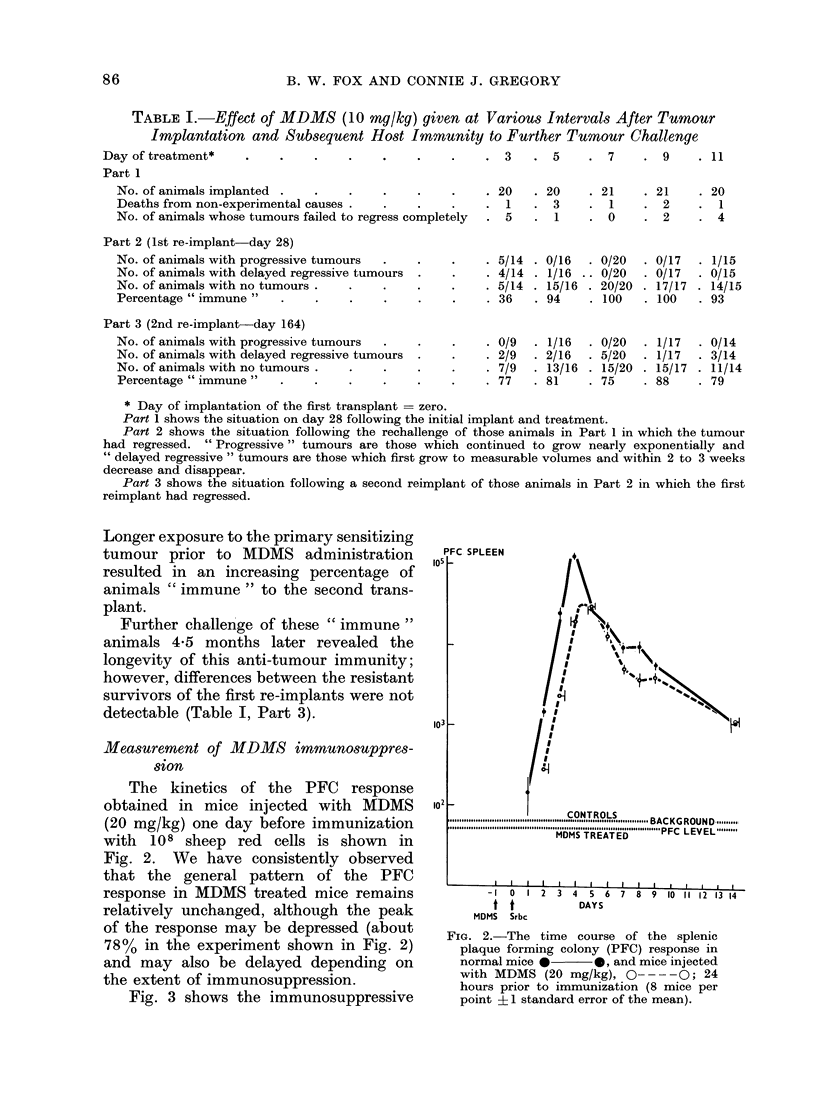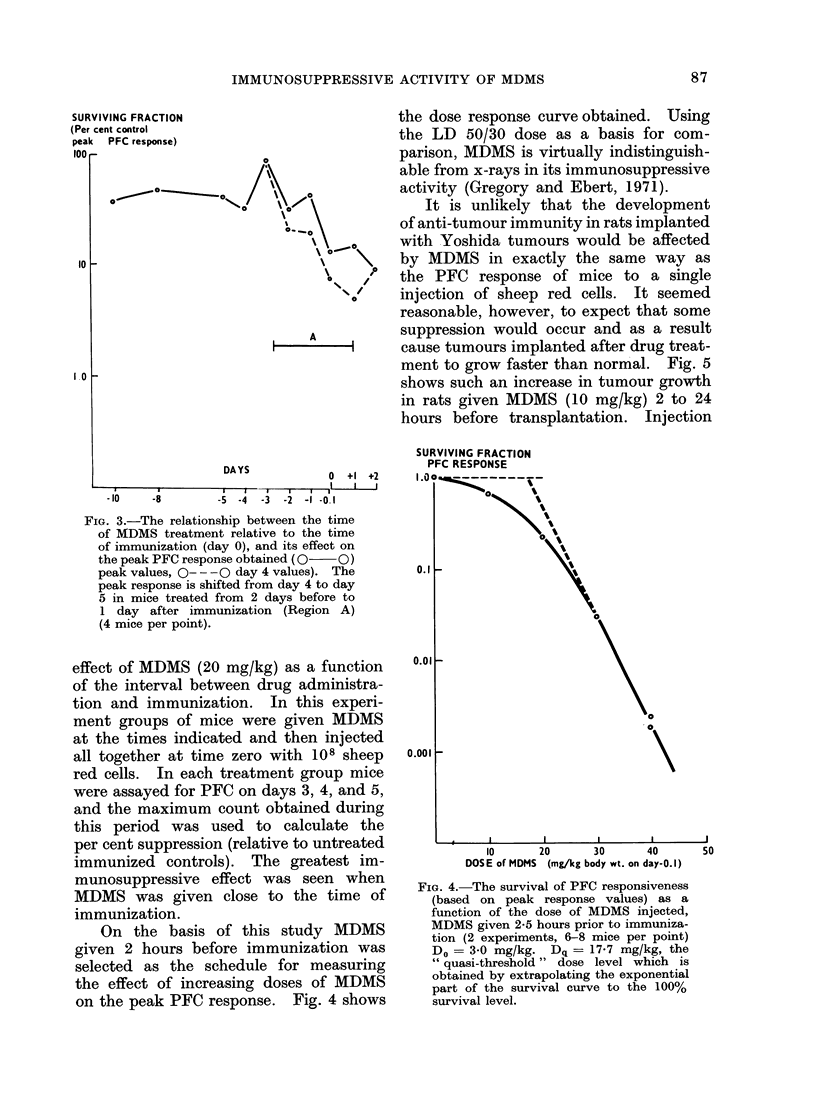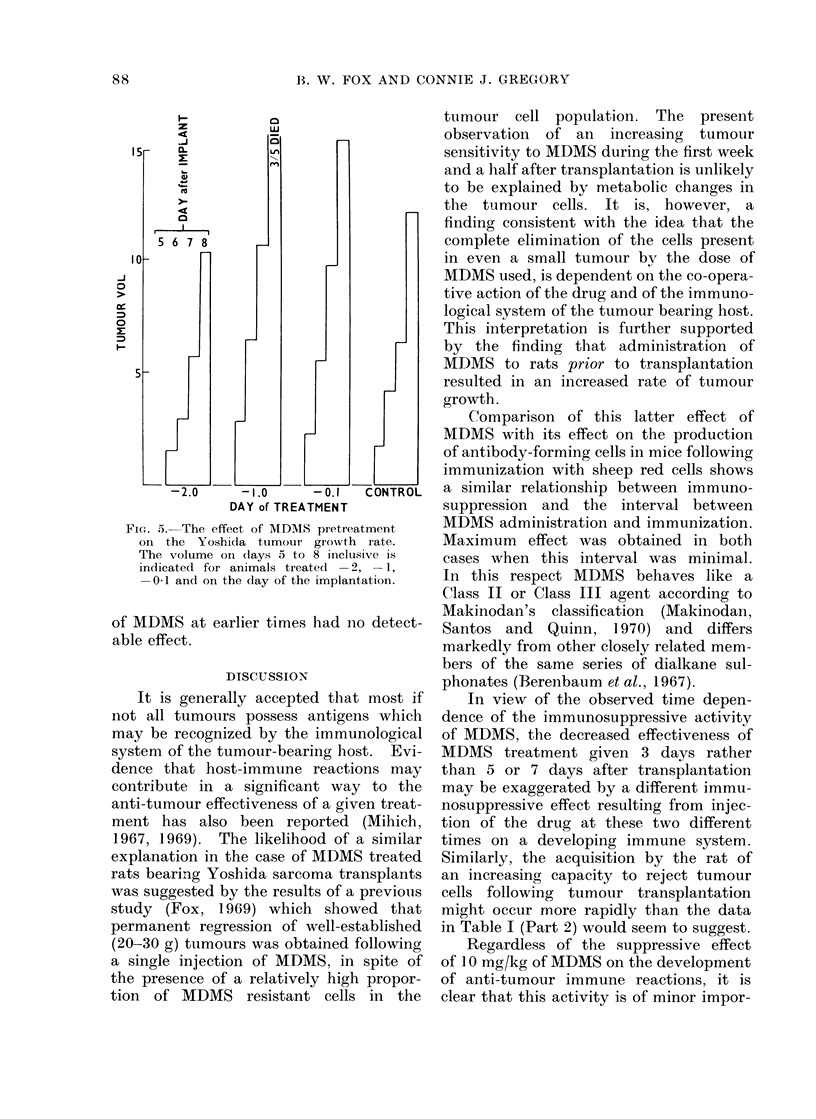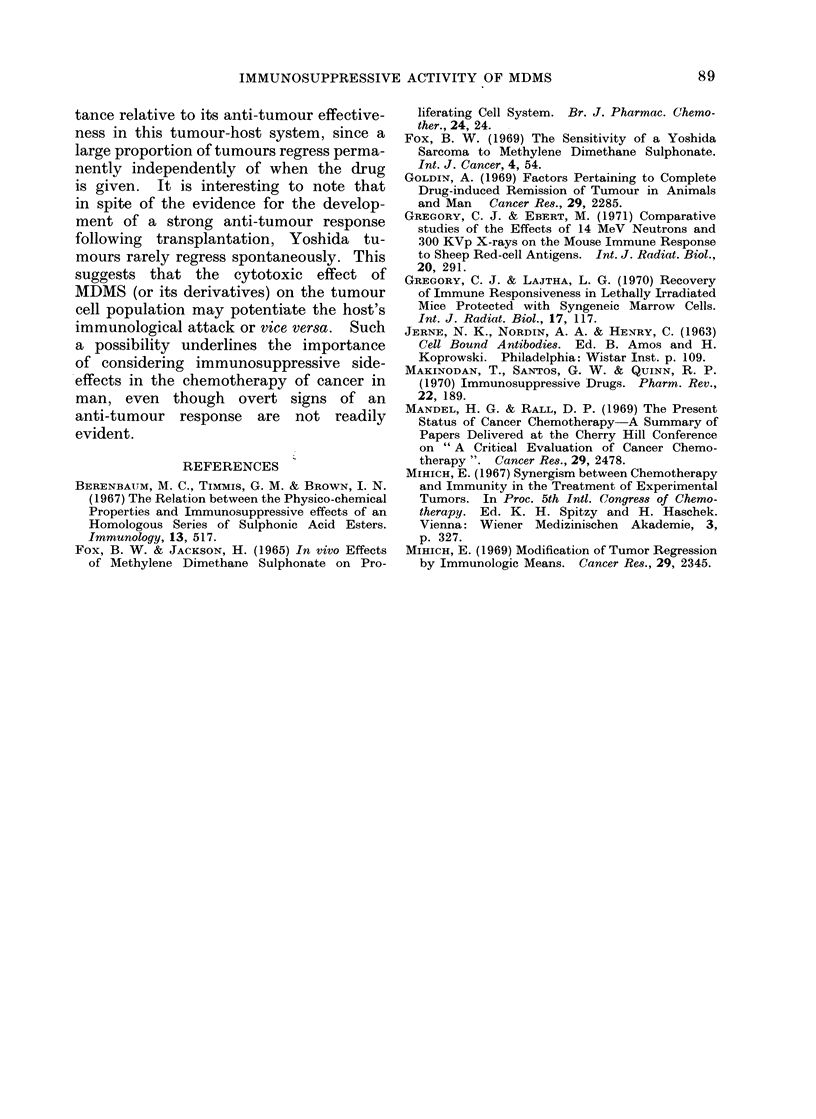# A Study of the Immunosuppressive Activity of Methylene Dimethane Sulphonate (MDMS) in Relation to its Effectiveness as an Anti-Tumour Agent

**DOI:** 10.1038/bjc.1972.13

**Published:** 1972-04

**Authors:** B. W. Fox, Connie J. Gregory

## Abstract

The anti-tumour action of methylene dimethane sulphonate (MDMS) has been further investigated in relation to its immunosuppressive properties. Following a dose of 10 mg/kg, the proportion of permanent regressions of Yoshida lymphosarcoma transplants is lower in animals treated during the first 5 days of tumour growth. Re-implants on day 28 to those animals in which regression of the tumour had occurred indicated that the immune response to the tumour increases during the first 7 days of tumour growth.

Studies of the effect of MDMS on the primary antibody-forming cell response of mice to sheep red cell antigens showed this drug to be an immunosuppressant comparable in strength to x-radiation. MDMS given to rats prior to tumour transplantation also acted as an immunosuppressant in this system resulting in an increased rate of tumour growth. For both responses the maximum immunosuppressive effect was obtained when the interval between drug administration and antigenic challenge was minimal.


*Br. J. Cancer* (1972) **26**, 84

### A STUDY OF THE IMMUNOSUPPRESSIVE ACTIVITY OF METHYLENE DIMETHANE SULPHONATE (MDMS) IN RELATION TO ITS EFFECTIVENESS AS AN ANTI-TUMOUR AGENT

B. W. FOX AND CONNIE J. GREGORY*

*Paterson Laboratories, Christie Hospital and Holt Radium Institute, Withington, Manchester M20 9BX*

Received for publication October 1971

**Summary.—The anti-tumour action of methylene dimethane sulphonate (MDMS) has been further investigated in relation to its immunosuppressive properties. Following a dose of 10 mg/kg, the proportion of permanent regressions of Yoshida lymphosarcoma transplants is lower in animals treated during the first 5 days of tumour growth. Re-implants on day 28 to those animals in which regression of the tumour had occurred indicated that the immune response to the tumour increases during the first 7 days of tumour growth.**

**Studies of the effect of MDMS on the primary antibody-forming cell response of mice to sheep red cell antigens showed this drug to be an immunosuppressant comparable in strength to x-radiation. MDMS given to rats prior to tumour transplantation also acted as an immunosuppressant in this system resulting in an increased rate of tumour growth. For both responses the maximum immunosuppressive effect was obtained when the interval between drug administration and antigenic challenge was minimal.**

METHYLENE dimethane sulphonate (MDMS) has been shown (Fox and Jackson, 1965; Fox, 1969) to exert an excellent anti-tumour action on the transplanted Yoshida lymphosarcoma in the Wistar rat. This tumour is equivalent to an allograft system, but the host reaction against the transplantation antigens, although great, does not normally reject the tumour which eventually kills the animal. Although tumour-specific antigens probably play a minor role in determining the host reaction against it, this system does provide a useful model system to study the contribution of host immunity to the chemotherapeutic effect of an anti-tumour drug (Mihich, 1969). The factors influencing drug-induced remission have been reviewed by Goldin (1969) and Mandel and Rall (1969).

The results of the present study indicate that the effectiveness of MDMS as an anti-tumour agent varies according to the level of immunity developed in the tumour-bearing host. We have also found that MDMS like other members of this series of bifunctional alkylating agents (Berenbaum, Timmis and Brown, 1967) possesses immunosuppressive properties as well as tumour cytotoxicity and the net effect of these two opposing activities in terms of tumour regression, depends on the time of MDMS administration relative to the time of tumour transplantation.

### MATERIALS AND METHODS

Methylene dimethane sulphonate was prepared as previously described (Fox and Jackson, 1965) and injected intraperitoneally in physiological saline (2–5 mg/ml). All solutions were prepared under chilled conditions immediately before use to avoid breakdown of the drug due to hydrolysis.

For the tumour growth studies, female Wistar rats (150–200 g, 8 to 10 weeks old)

* Present address: Department of Medical Biophysics, 500 Sherbourne Street, Toronto 5, Canada.

fed on a standard diet based on the Scottish N.E. Agricultural Society recommendations and water *ad libitum* were used. Small pieces of tumour tissue (approximately 5 $mm^3$) were transplanted subcutaneously by trochar implantation into the flank of the animal. The tumour volume was measured as previously described (Fox, 1969) on successive days of growth.

FIG. 1.—The effect on growth of the Yoshida sarcoma of MDMS (10 mg/kg) administered intraperitoneally on days 3, 5, 7, 9, 11 (A to E) following transplantation. The letters above the abscissa indicate the corresponding growth curves. The ordinate is a caliper derived volume in ml.

For studies of the anti-sheep red cell response, 12 to 14 week old (C3H × AKR) F1 male mice were given a single intravenous injection of $10^8$ washed sheep red cells (Burroughs Wellcome and Co.). Cells producing 19S haemolytic anti-sheep red cell antibody (PFC) were detected using the method of Jerne, Nordin and Henry (1963). Spleen cells were suspended in appropriate volumes of cold Eagle's medium (Burroughs Wellcome and Co.) buffered with 0·002M Tris-HCl and assayed individually in plates of agarose (L'Industrie Biologique Francaise, France) made up in the same medium. Two or 3 replicate plates were prepared from each spleen and the geometric mean PFC response per spleen calculated as described previously (Gregory and Lajtha, 1970).

### RESULTS

### *Action of MDMS on the Yoshida Tumour at Different Stages of Growth and its Effect on Subsequent Host Susceptibility to Further Tumour Challenge*

A large number of rats were transplanted with Yoshida tumour tissue, mixed at random and then separated into 5 groups of approximately 20 animals per group. The groups were then given MDMS (10 mg/kg) at 3, 5, 7, 9, and 11 days respectively, post-tumour transplantation and tumour volumes were measured up to the 28th day. The pattern of tumour regression obtained in each group was similar (Fig. 1). However, only those animals that showed no regrowth of the tumour following the initial regression were used in the construction of this figure. The number of animals in each group excluded because of tumour regrowth is indicated in Table I (Part 1). It can be seen that, paradoxically, animals treated 3 days after transplantation were *less* likely to show complete regression of their tumours than animals with much larger tumours (up to more than 10 times larger) treated on the 5th to 9th day. To determine whether this variation in the effectiveness of MDMS treatment could be related to a corresponding variation in the level of the anti-tumour immunity developed in the host, the tumour-free survivors in each group were tested for their ability to reject a second Yoshida tumour transplanted 28 days after receiving the first transplant. Only 36% of the animals whose initial tumours were allowed to grow for 3 days prior to MDMS treatment were sufficiently sensitized to prevent a second transplant from becoming even temporarily established (Table I, Part 2).

TABLE I.—*Effect of MDMS (10 mg/kg) given at Various Intervals After Tumour Implantation and Subsequent Host Immunity to Further Tumour Challenge*

| Day of treatment* | 3 | 5 | 7 | 9 | 11 |
|---|---|---|---|---|---|
| Part 1 | | | | | |
| No. of animals implanted | 20 | 20 | 21 | 21 | 20 |
| Deaths from non-experimental causes | 1 | 3 | 1 | 2 | 1 |
| No. of animals whose tumours failed to regress completely | 5 | 1 | 0 | 2 | 4 |
| Part 2 (1st re-implant—day 28) | | | | | |
| No. of animals with progressive tumours | 5/14 | 0/16 | 0/20 | 0/17 | 1/15 |
| No. of animals with delayed regressive tumours | 4/14 | 1/16 | 0/20 | 0/17 | 0/15 |
| No. of animals with no tumours | 5/14 | 15/16 | 20/20 | 17/17 | 14/15 |
| Percentage " immune " | 36 | 94 | 100 | 100 | 93 |
| Part 3 (2nd re-implant—day 164) | | | | | |
| No. of animals with progressive tumours | 0/9 | 1/16 | 0/20 | 1/17 | 0/14 |
| No. of animals with delayed regressive tumours | 2/9 | 2/16 | 5/20 | 1/17 | 3/14 |
| No. of animals with no tumours | 7/9 | 13/16 | 15/20 | 15/17 | 11/14 |
| Percentage " immune " | 77 | 81 | 75 | 88 | 79 |

* Day of implantation of the first transplant = zero.
*Part* 1 shows the situation on day 28 following the initial implant and treatment.
*Part* 2 shows the situation following the rechallenge of those animals in Part 1 in which the tumour had regressed. " Progressive " tumours are those which continued to grow nearly exponentially and " delayed regressive " tumours are those which first grow to measurable volumes and within 2 to 3 weeks decrease and disappear.
*Part* 3 shows the situation following a second reimplant of those animals in Part 2 in which the first reimplant had regressed.

Longer exposure to the primary sensitizing tumour prior to MDMS administration resulted in an increasing percentage of animals " immune " to the second transplant.

Further challenge of these " immune " animals 4·5 months later revealed the longevity of this anti-tumour immunity; however, differences between the resistant survivors of the first re-implants were not detectable (Table I, Part 3).

*Measurement of MDMS immunosuppression*

The kinetics of the PFC response obtained in mice injected with MDMS (20 mg/kg) one day before immunization with $10^8$ sheep red cells is shown in Fig. 2. We have consistently observed that the general pattern of the PFC response in MDMS treated mice remains relatively unchanged, although the peak of the response may be depressed (about 78% in the experiment shown in Fig. 2) and may also be delayed depending on the extent of immunosuppression.

Fig. 3 shows the immunosuppressive

FIG. 2.—The time course of the splenic plaque forming colony (PFC) response in normal mice ●——●, and mice injected with MDMS (20 mg/kg), ○- - - -○; 24 hours prior to immunization (8 mice per point ±1 standard error of the mean).

FIG. 3.—The relationship between the time of MDMS treatment relative to the time of immunization (day 0), and its effect on the peak PFC response obtained (O——O) peak values, O– – –O day 4 values). The peak response is shifted from day 4 to day 5 in mice treated from 2 days before to 1 day after immunization (Region A) (4 mice per point).

effect of MDMS (20 mg/kg) as a function of the interval between drug administration and immunization. In this experiment groups of mice were given MDMS at the times indicated and then injected all together at time zero with $10^8$ sheep red cells. In each treatment group mice were assayed for PFC on days 3, 4, and 5, and the maximum count obtained during this period was used to calculate the per cent suppression (relative to untreated immunized controls). The greatest immunosuppressive effect was seen when MDMS was given close to the time of immunization.

On the basis of this study MDMS given 2 hours before immunization was selected as the schedule for measuring the effect of increasing doses of MDMS on the peak PFC response. Fig. 4 shows the dose response curve obtained. Using the LD 50/30 dose as a basis for comparison, MDMS is virtually indistinguishable from x-rays in its immunosuppressive activity (Gregory and Ebert, 1971).

It is unlikely that the development of anti-tumour immunity in rats implanted with Yoshida tumours would be affected by MDMS in exactly the same way as the PFC response of mice to a single injection of sheep red cells. It seemed reasonable, however, to expect that some suppression would occur and as a result cause tumours implanted after drug treatment to grow faster than normal. Fig. 5 shows such an increase in tumour growth in rats given MDMS (10 mg/kg) 2 to 24 hours before transplantation. Injection

FIG. 4.—The survival of PFC responsiveness (based on peak response values) as a function of the dose of MDMS injected, MDMS given 2·5 hours prior to immunization (2 experiments, 6–8 mice per point) $D_0$ = 3·0 mg/kg. $D_q$ = 17·7 mg/kg, the " quasi-threshold " dose level which is obtained by extrapolating the exponential part of the survival curve to the 100% survival level.

FIG. 5.—The effect of MDMS pretreatment on the Yoshida tumour growth rate. The volume on days 5 to 8 inclusive is indicated for animals treated −2, −1, −0·1 and on the day of the implantation.

of MDMS at earlier times had no detectable effect.

DISCUSSION

It is generally accepted that most if not all tumours possess antigens which may be recognized by the immunological system of the tumour-bearing host. Evidence that host-immune reactions may contribute in a significant way to the anti-tumour effectiveness of a given treatment has also been reported (Mihich, 1967, 1969). The likelihood of a similar explanation in the case of MDMS treated rats bearing Yoshida sarcoma transplants was suggested by the results of a previous study (Fox, 1969) which showed that permanent regression of well-established (20–30 g) tumours was obtained following a single injection of MDMS, in spite of the presence of a relatively high proportion of MDMS resistant cells in the tumour cell population. The present observation of an increasing tumour sensitivity to MDMS during the first week and a half after transplantation is unlikely to be explained by metabolic changes in the tumour cells. It is, however, a finding consistent with the idea that the complete elimination of the cells present in even a small tumour by the dose of MDMS used, is dependent on the co-operative action of the drug and of the immunological system of the tumour bearing host. This interpretation is further supported by the finding that administration of MDMS to rats *prior* to transplantation resulted in an increased rate of tumour growth.

Comparison of this latter effect of MDMS with its effect on the production of antibody-forming cells in mice following immunization with sheep red cells shows a similar relationship between immunosuppression and the interval between MDMS administration and immunization. Maximum effect was obtained in both cases when this interval was minimal. In this respect MDMS behaves like a Class II or Class III agent according to Makinodan's classification (Makinodan, Santos and Quinn, 1970) and differs markedly from other closely related members of the same series of dialkane sulphonates (Berenbaum *et al.*, 1967).

In view of the observed time dependence of the immunosuppressive activity of MDMS, the decreased effectiveness of MDMS treatment given 3 days rather than 5 or 7 days after transplantation may be exaggerated by a different immunosuppressive effect resulting from injection of the drug at these two different times on a developing immune system. Similarly, the acquisition by the rat of an increasing capacity to reject tumour cells following tumour transplantation might occur more rapidly than the data in Table I (Part 2) would seem to suggest.

Regardless of the suppressive effect of 10 mg/kg of MDMS on the development of anti-tumour immune reactions, it is clear that this activity is of minor importance relative to its anti-tumour effectiveness in this tumour-host system, since a large proportion of tumours regress permanently independently of when the drug is given. It is interesting to note that in spite of the evidence for the development of a strong anti-tumour response following transplantation, Yoshida tumours rarely regress spontaneously. This suggests that the cytotoxic effect of MDMS (or its derivatives) on the tumour cell population may potentiate the host's immunological attack or *vice versa*. Such a possibility underlines the importance of considering immunosuppressive side-effects in the chemotherapy of cancer in man, even though overt signs of an anti-tumour response are not readily evident.

### REFERENCES

BERENBAUM, M. C., TIMMIS, G. M. & BROWN, I. N. (1967) The Relation between the Physico-chemical Properties and Immunosuppressive effects of an Homologous Series of Sulphonic Acid Esters. *Immunology*, **13**, 517.

FOX, B. W. & JACKSON, H. (1965) *In vivo* Effects of Methylene Dimethane Sulphonate on Proliferating Cell System. *Br. J. Pharmac. Chemother.*, **24**, 24.

FOX, B. W. (1969) The Sensitivity of a Yoshida Sarcoma to Methylene Dimethane Sulphonate. *Int. J. Cancer*, **4**, 54.

GOLDIN, A. (1969) Factors Pertaining to Complete Drug-induced Remission of Tumour in Animals and Man *Cancer Res.*, **29**, 2285.

GREGORY, C. J. & EBERT, M. (1971) Comparative studies of the Effects of 14 MeV Neutrons and 300 KVp X-rays on the Mouse Immune Response to Sheep Red-cell Antigens. *Int. J. Radiat. Biol.*, **20**, 291.

GREGORY, C. J. & LAJTHA, L. G. (1970) Recovery of Immune Responsiveness in Lethally Irradiated Mice Protected with Syngeneic Marrow Cells. *Int. J. Radiat. Biol.*, **17**, 117.

JERNE, N. K., NORDIN, A. A. & HENRY, C. (1963) *Cell Bound Antibodies*. Ed. B. Amos and H. Koprowski. Philadelphia: Wistar Inst. p. 109.

MAKINODAN, T., SANTOS, G. W. & QUINN, R. P. (1970) Immunosuppressive Drugs. *Pharm. Rev.*, **22**, 189.

MANDEL, H. G. & RALL, D. P. (1969) The Present Status of Cancer Chemotherapy—A Summary of Papers Delivered at the Cherry Hill Conference on " A Critical Evaluation of Cancer Chemotherapy ". *Cancer Res.*, **29**, 2478.

MIHICH, E. (1967) Synergism between Chemotherapy and Immunity in the Treatment of Experimental Tumors. In *Proc. 5th Intl. Congress of Chemotherapy*. Ed. K. H. Spitzy and H. Haschek. Vienna: Wiener Medizinischen Akademie, **3**, p. 327.

MIHICH, E. (1969) Modification of Tumor Regression by Immunologic Means. *Cancer Res.*, **29**, 2345.